# Echocardiographic Evaluation of the Effects of a Single Bolus of Erythropoietin on Reducing Ischemia-Reperfusion Injuries during Coronary Artery Bypass Graft Surgery; A Randomized, Double-Blind, Placebo-Control Study

**Published:** 2014-03

**Authors:** Shervin Ziabakhsh-Tabary, Rozita Jalalian, Farzad Mokhtari-Esbuie, Mohammad Reza Habibi

**Affiliations:** 1 Department of Cardiac Surgery, Fatemeh Zahra Hospital, Mazandaran University of Medical Sciences, Sari, Iran;; 2 Department of Cardiovascular, Fatemeh Zahra Hospital, Mazandaran University of Medical Sciences, Sari, Iran;; 3 General Physician, Fatemeh Zahra Hospital, Mazandaran University of Medical Science, Sari, Iran;; 4 Department of Anesthesiology, Fatemeh Zahra Hospital, Mazandaran University of Medical Sciences, Sari, Iran

**Keywords:** Erythropoietin, Ischemia, Reperfusion injury, Coronary artery bypass graft

## Abstract

**Background: **Erythropoietin (EPO) is known as a regulating hormone for the production of red blood cells, called erythropoiesis. Some studies have shown that EPO exerts some non-hematopoietic protective effects on ischemia-reperfusion injuries in myocytes. Using echocardiography, we evaluated the effect of EPO infusion on reducing ischemia-reperfusion injuries and improvement of the cardiac function shortly after coronary artery bypass graft surgery (CABG).

**Methods: **Forty-three patients were recruited in this study and randomly divided into two groups: the EPO group, receiving standard medication and CABG surgery plus EPO (700 IU/kg), and the control group, receiving standard medication and CABG surgery plus normal saline (10 cc) as placebo. The cardiac function was assessed through echocardiography before as well as at 4 and 30 days after CABG.

**Results:** Echocardiography indicated that the ejection fraction had no differences between the EPO and control groups at 4 days (47.05±6.29 vs. 45.90±4.97; P=0.334) and 30 days after surgery (47.27±28 vs. 46.62±5.7; P=0.69). There were no differences between the EPO and control groups in the wall motion score index at 4 (P=0.83) and 30 days after surgery (P=0.902). In the EPO group, there was a reduction in left ventricular end-systolic and end-diastolic diameters (LVESD and LVEDD, respectively), as compared to the control group.

**Conclusion: **Our results indicated that perioperative exogenous EPO infusion could not improve the ventricular function and wall motion index in the immediate post-CABG weeks. Nevertheless, a reduction in LVEDD and LVESD at 4 days and 30 days after CABG in the EPO group, by comparison with the control group, suggested that EPO correlated with a reduction in the remodeling of myocytes and reperfusion injuries early after CABG.

**Trial Registration Number: **138809102799N1

## Introduction

Erythropoietin (EPO) is a glycoprotein hormone produced by the kidney and plays a key role in hematopoiesis.^1^ In addition to these hematopoietic effects, EPO exerts non-hematopoietic effects on some tissues like the brain,^2^ kidney,^3^ retina,^4^ and muscles.^5^ Moreover, both ventricular myocytes and endothelial cells have EPO receptors.^6^ EPO wields its protective effects on myocardial cells via different pathways, including stimulation of neovascularization, activation of the PI3K and 2.1 ERK pathways,^7^^,^^8^ and synthesis stimulation of endothelial progenitor cells from the bone marrow.^9^^,^^10^

Coronary artery bypass graft surgery (CABG), an important treatment modality in ischemic patients, increases myocardial perfusion and ejection fraction (EF) in patients with coronary artery diseases.^11^ Although the rapid reperfusion by CABG significantly reduces mortality and morbidity,^12^ this reperfusion paradoxically may contribute to myocardial stunning injuries and/or death after CABG.^13^^,^^14^ Therefore, new treatment modalities should focus on decreasing damage after reperfusion.

In addition to the protective effect of EPO on myocardial ischemia, studies on animal models have shown that EPO can also reduce reperfusion tissue injuries.^15^^-^^17^ Studies on human models have, however, proved somewhat controversial.^18^^,^^19^ While some authors have reported that EPO can reduce ischemia-reperfusion injuries in the myocardium and posited the possible mechanism for this action,^20^^,^^21^ others such as Mocini et al.^19^ in a different model, performed on patients having undergone CABG, have maintained that EPO has no association with a reduction in the levels of myocardial biomarkers (troponin I and CKMB) after CABG. The latter group of authors justify their conclusions by arguing that EPO induces tissue protection with anti-apoptotic mechanism. Nonetheless, these authors assessed the effects of EPO by two indicators of necrosis, namely troponin I and CKMB.

The left ventricular (LV) function is usually described in terms of the EF. Given the aforementioned controversy in the results of the studies conducted hitherto and the importance of injuries after ischemia and reperfusion in CABG, we designed a double-blind, controlled trial to evaluate the protective effects of EPO on post-CABG reperfusion injuries through assessment of echocardiographic parameters before and after CAGB.

## Patients and Methods

*Study Design*

**Figure 1:**
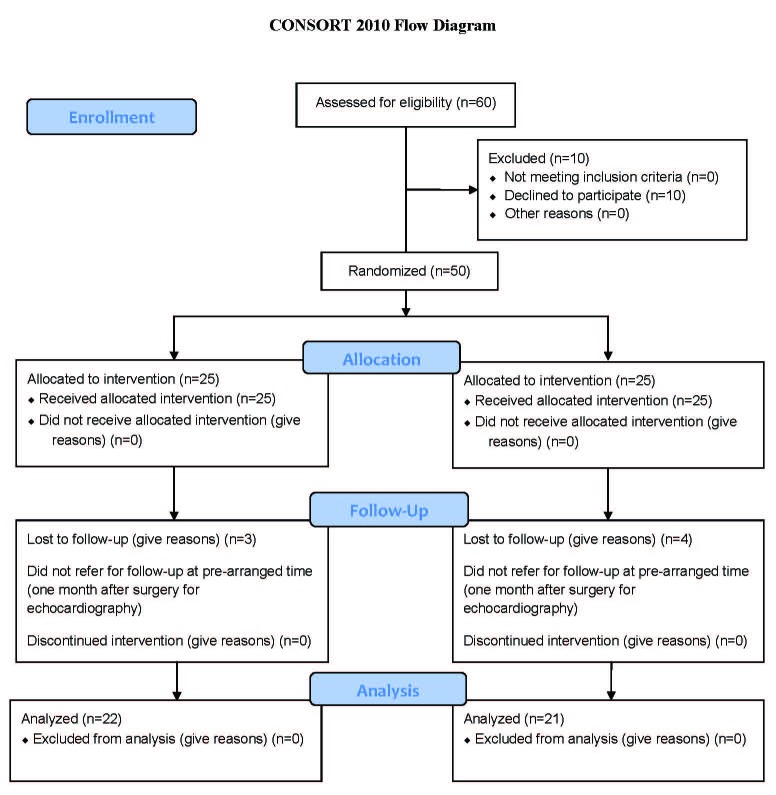
The patient’s consort flow chart is illustrated above.

In this randomized, double-blind, clinical trial, the study population was comprised of all patients that were referred to Fatemeh Zahra Hospital (Sari, Iran) for elective CABG between September 2010 and October 2011. According to previous studies and statistical analyses, 50 patients who met the inclusion criteria and passed the exclusion filter were randomly divided into two groups. The case and control lists were blinded to the patients and the cardiac surgeon. Seven patients failed to refer for their third echocardiographic examination at a pre-arranged time (one month after surgery) and were, thus, removed from the study. Consequently, 43 patients remained in the evaluation. The patients’ CONSORT flow diagram is depicted in figure 1.

The patients in the EPO group were treated with common medical therapies and CABG plus an intravenous infusion of 700 IU/kg of EPO (PD Poietin, Puyesh Daroo Olfactory, Iran), exactly 5 minutes after the termination of cross-clamp at the start of reperfusion. The patients in the control group were treated with common medical therapies and CABG plus 10 cc of normal saline as placebo. All the surgical operations were performed by the same cardiac surgeon and anesthesiologist. A technician of anesthesiology was provided with the list of the patients of the EPO and control groups and was responsible for the injection of EPO or saline as placebo. The trial was parallel. The study method was approved by the institutional Ethics Committee, and written informed consent was obtained from all the patients. 

The inclusion criterion was revascularization requirement according to angiographic evidence. The exclusion criteria were comprised of history of myocardial infarction in the past 3 months, previous myocardial trauma or major surgery in the past 3 months, EF<30%, Cr>2.5, receiving streptokinase or previous reperfusion treatments, EPO intake in the recent 6 months, and polycythemia. 

Transthoracic echocardiography (using Vivid S5 Machine) with the Simpson method and also Doppler echocardiography were performed in all the patients at three times: 1) one or 2 days before surgery; 2) four days after surgery; and 3) thirty days after CABG. Regional wall motion was evaluated using the 16-segment model as recommended by the American Society of Echocardiography. Other variables that were measured 2 or 3 days before surgery included age, gender, body mass index (BMI), blood pressure, cholesterol, BUN, Cr, BS, Hgb, Hct, plt, Retic, Na, K (Pars Test kits), EF, and cross-clamping time.

*Statistical Analysis*

The patients were matched for demographic characteristics. Group differences for the continuous variables were examined using the *t* test. The data distributions were checked using the Kolmogorov-Smirnov test. The Mann-Whitney test was performed for the data that did not follow normal distribution. As regards the categorical variables, group differences were examined using the Chi-square test. The results were considered statistically significant when P<0.05. The statistical analyses were conducted with SPSS software (version 16).

## Results

There were no differences between the EPO and control groups in terms of the number of impaired vessels (2.27±0.787 vs. 2.29±0.784; P=0.863) and age (59.73±7.73 vs. 62.57±8.6; P=1.878). Table 1 presents further information on the patients in the two groups.

**Table 1:** Primary characteristics of the patients

	**EPO group**	**Control group**	**P value**
Number	22	21	
Gender	13	8	0.1
Age(year)	59.73±7.73	62.57±8.60	1.878
Smoking	14	13	0.4
Diabetes history	8	10	0.9
BMI(kg/m^ 2 ^)	25.82±1.83	24.36±2.12	0.009
Creatinine (mg/dl)	0.94±0.18	0.86±0.27	0.149
Impaired vessels(n)	2.27±0.787	2.29±0.784	0.863
EF before operation(n)	46.36±8.04	45.90±8.41	0.178
Hgb (g/dl)	12.64±2.10	13±1.20	0.955
Retic (%)	0.85±0.33	0.61±0.25	0.166
Na (mEq/L)	141.36±1.98	140.95±4.11	0.67
K (mEq/L)	4.20±0.35	4.45±0.44	0.594
FBS (mg/dl)	132.68±55.19	158.95±79.98	0.132
Pack cell (n)	1±1.06	0.48±0.68	0.021
Graft (n)	3.14±0.88	3.38±0.74	0.33
Pump time (min)	78.21±18.8	79.76±12.25	0.6
Cross-clamping time (min)	50.95±10.85	53.86±9.13	0.08

EPO: Erythropoietin; BMI: Body mass index; EF: Ejection fraction

As is shown in table 2, there were no significantly differences between the EPO and control groups regarding the EF at 4 days after surgery (47.05±6.29 vs. 45.90±4.97; P=0.334) and also 30 days after surgery (47.27±28 vs. 46.62±5.7; P=0.69).

**Table 2:** Patients’ EF before and after CABG in both groups

	**EPO group**	**Control group**	**P value**
EF Before surgery	46.36±8.04	45.90±6.42	0.178
EF 4 days after surgery	47.05±6.29	45.90±4.97	0.334
EF 30 days after surgery	47.27±28	46.62±5.7	0.69

EF: Ejection fraction; EPO: Erythropoietin; CABG: Coronary artery bypass graft

**Figure 2:**
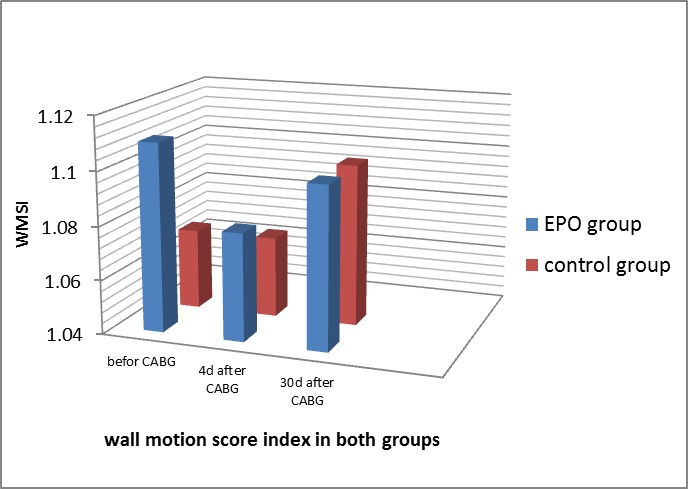
Wall motion score index before and after coronary artery bypass graft surgery in both groups.

The mean level of the wall motion score index (WMSI) also had no differences between the EPO and control groups at 4 days after surgery (1.08±0.09 vs. 1.07±0.10; P=0.83) and also 30 days after surgery (1.10±0.13 vs. 1.10±0.16; P=0.902) (figure 2). The mean levels of left ventricular end-diastolic diameter (LVEDD) and left ventricular end-systolic diameter (LVESD) are shown in table 3.

**Table 3:** Patients’ echocardiographic parameters in both groups

	**EPO group**	**Control group**	**P value**
WMSI before CABG	1.11±0.12	1.07±0.10	0.15
WMSI 4 days after CABG	1.08±0.09	1.07±0.10	0.83
WMSI 30 days after CABG	1.10±0.13	1.10±0.16	0.902
LVEDD before CABG	5.09± 0.70	4.68±0.94	0.314
LVEDD 4 days after CABG	4.86±0.74	4.73±0.59	0.436
LVEDD 30 days after CABG	4.95±0.68	4.79±0.61	0.434
LVESD before CABG	3.72±0.79	3.63±0.84	0.825
LVESD 4 days after CABG	3.53±0.75	3.67±0.54	0.230
LVESD 30 days after CABG	3.55±0.71	3.77±0.77	0.876

EPO: Erythropoietin; LVEDD: Left ventricular end-diastolic diameter; LVESD: Left ventricular end-systolic diameter

S showed a significant rise at the 30^th^ postoperative day in the EPO group (5.59+0.90 vs. 6.68+1.524; P=0.024), while it had a drop in the control group (6.33+1.11 vs. 5.61+1.07; P=0.015). Also, whereas E/A (1.02+0.83 vs. 0.95+0.28; P=0.717) and E/E’ (0.17+0.19 vs. 0.14+0.14; P=0.490) non-significantly decreased at 30 days after surgery in the EPO group, E/A (0.70+0.15 vs. 0.91+0.28; P=0.004) and E/E’ (0.10+0.03 vs. 0.12+0.04; P=0.188) significantly increased in the control group at 30 days after surgery.

No important complications such as myocardial infarction, mean arterial pressure rise, and thromboembolic events were seen in the patients in the EPO group during surgery and in the first postoperative month. 

## Discussion

New articles have mentioned that the early post-CABG period is suboptimal for the estimation of the ventricular function due to perioperative ischemia and reperfusion injuries, which can negatively affect the contractile function.^22^ The present study evaluated the effect of a single bolus of EPO on the first 4 weeks after CABG.

The LV function is usually described in terms of the EF.^23^ In the present study, there were no significant differences between the EPO and control groups with respect to the EF at 4 days and also 30 days after surgery, which means that EPO had no effect on improving the ventricular function in the first 4 weeks following CABG.

It is not clear whether or not EF is the most meaningful index of the LV function in ischemic and infarct situations. A low EF may be caused by poor contractile function due to extensive myocardial damage or continuing ischemia. One study reported that end-systolic volume or end-diastolic volume might be better than the EF in the prediction of prognosis.^24^ Some authors have argued that a significant dilatation of the LV, commencing immediately after coronary occlusion in rats, can produce an increase in LVEDP and a diminished slope of LV pressure versus time. These changes noticeably increase myocardial wall stress. What is also of note is that this process leads to an increase in the ventricular diameter and volume and that these changes typically establish an advantageous adaptation while cardiac ischemia or infarction is occurring in the acute phase by maintaining critical pump function. Nonetheless, such remodeling unavoidably results in inefficient pump function, which can bring about hemodynamic deterioration.^20^ LVEDD and LVESD are two extremely valuable parameters for the estimation of the LV function. The literature contains a large number of studies that drew upon these parameters as useful and important parameters for assessing the LV remodeling and LV function.^25^^,^^26^

In comparison with our control group, EPO was correlated with a slight reduction in LVEDD and LVESD at 4 days and also 30 days after surgery from baseline; the difference between the two groups, however, did not constitute statistical significance. This finding means that EPO infusion can reduce reperfusion injuries and myocyte remodeling and improve prognosis in ischemic situations such as CABG.

Our results showed that, as compared to the control group, EPO had no effect on the reduction in the WMSI at 4 days and also 30 days after surgery. The WMSI is a good indicator of ventricular septum dysfunction, and echocardiographic determination of the wall motion is a useful tool for observing the LV function.^24^ In the present study, the WMSI had no differences between the two groups at 4 days and also 30 days after surgery, denoting that the administration of EPO during CABG had no effect on the reduction of the remodeling and stunning of the ventricular septum at 4 days and 30 days after surgery. In addition, it is possible that a long-term evaluation of the effectiveness of EPO would have yielded different results. Previous studies did not utilize the WMSI, LVESD, and LVEDD to evaluate the protective effects of EPO against ischemia-reperfusion injuries postoperatively, which adds further significance to our results. As a case in point, Mocini et al.^19^ evaluated EPO efficacy by measuring troponin I and CKMB levels.

We also assessed the diastolic function by measuring specific echocardiographic parameters such as S, E, and E’. Our results showed that the diastolic function exhibited improvement one month after surgery in the EPO group. (E/A and E/E’ showed a drop one month after surgery, while they had a rise in the control group.) Also, the rise of S in the EPO group at one month postoperatively was allied to an improvement in the systolic function.

We examined the effect of EPO in the first 4 post-CABG weeks. Time needed for the LV function improvement depends on the level of degeneration and connective tissue proliferation. Some studies have found no alteration or deterioration in segmental wall motion within the first week postoperatively and showed myocardial improvement by assessing the WMSI and LVEF at 3 to 6 months after surgery.^27^^-^^29^ In contrast, other studies have reported improvement in myocardial contractibility within the first intraoperative days or within the first postoperative weeks.^24^^,^^27^^,^^30^ Further and long-term follow-up is required in these patients to determine whether EPO has efficacy in the WMSI changes and ventricular function after CABG.

It is worthy of note that most of our patients had EF>30% and only 6 patients had EF<30%. As a result, it is possible that the efficacy of EPO on the ventricular function in patients with lower EF is higher than in patients with acceptable EF. We suggest that future studies recruit patients with lower EF to examine the effect of EPO on these patients.

Recent studies have disagreed about the effective dosage of EPO for lessening the damage of ischemia-reperfusion. Animal experimental models have used higher doses than human experimental models. Of the former group, the results of a study by L. Javadi^16^ showed that 5000 IU/kg of EPO could reduce the infarct area, minimize cell damage, and reduce myocytes apoptosis. In Lipsic at al’s.^31^ study, the same dosage was used and similar results were obtained. Salient among the human experimental models, with lower doses of EPO, is a case-control study by Mocini et al.^19^ who used 40000 IU of EPO and found no differences in troponin I and CKMB levels in both EPO and control groups; the authors concluded that there might be a correlation between this result and the EPO dosage. In the present study, we used 700 IU/kg of PD-Poietin, which was estimated to be equal to the EPO dosage in the Mocini et al.^19^ study.

The optimal time for EPO infusion has yet to be fully elucidated. In some studies, EPO was infused 24 hours before ischemia and reperfusion.^16^^,^^18^ In Lipsic et al’s.^31^ study, the effectiveness of EPO was measured according to the rate of apoptosis and percentage of active caspase-3 enzyme, and subsequently the time of EPO prescription was evaluated; it was concluded that the best time for EPO infusion was after the onset of reperfusion post ischemia during surgery. In Mocini et al’s.^19^ study, EPO was injected in the immediate pre-surgical period. In our study, we used EPO at the start of tissue reperfusion after aorta clamping. Therefore, as was mentioned before, further research is required to clearly determine the optimal time for EPO prescription in human experiments. It is likely that the resultant controversy about the effectiveness of EPO in this study was due to the time of infusion; we might have achieved more optimal results if we had injected EPO 24 hours before CABG.

The cost of EPO treatment and the uncertain outcome in patients’ life expectancy may be the reasons why surgeons hesitate to use EPO more frequently in their cardiac surgeries. As for the present study, the slight discrepancy in the results obtained from the analysis of the different parameters might be due to the insufficient number of our patients. Our results can indeed be drawn upon by future studies with larger sample populations probing into the effectiveness of EPO in patients candidated for CABG. 

## Conclusion

Our data suggest that perioperative exogenous EPO infusion cannot improve the ventricular function and WMSI in the first weeks after surgery. A reduction in the levels of LVEDD and LVESD at 4 days and 30 days after CABG in the EPO group, by comparison with the control group, indicated that EPO was correlated with the reduction in myocyte remodeling and reperfusion injuries early after CABG.

## Suggestion

We need more long-term evaluation to clearly determine whether EPO prescription during surgery can increase the survival rate and LV function. In light of the results of the present study, we recommend that future studies in this domain recruit larger numbers of patients, especially those with lower EF.
